# RPN2 is targeted by miR-181c and mediates glioma progression and temozolomide sensitivity via the wnt/β-catenin signaling pathway

**DOI:** 10.1038/s41419-020-03113-5

**Published:** 2020-10-22

**Authors:** Jikui Sun, Quanfeng Ma, Banban Li, Chen Wang, Lidong Mo, Xuebin Zhang, Fan Tang, Qiong Wang, Xiaoling Yan, Xiuhua Yao, Qiaoli Wu, Chang Shu, Jinbiao Xiong, Weijia Fan, Jinhuan Wang

**Affiliations:** 1 School of Medicine, Nankai University, 94 Weijin Road, Tianjin, 300071 China; 2 Tianjin Cerebral Vascular and Neural Degenerative Disease Key Laboratory, Tianjin Neurosurgical Institute, Department of Neurosurgery, Tianjin Huanhu Hospital, Tianjin, 300350 China; 3 Department of Hematology, Taian Central Hospital, Taian, 271000 China; 4 Pathology Department, Tianjin Huanhu Hospital, Tianjin, 300350 China; 5 Tianjin Neurological Institute, Tianjin Medical University General hospital, Tianjin, 300052 China

**Keywords:** CNS cancer, miRNAs

## Abstract

Accumulating evidence indicates that the dysregulation of the miRNAs/mRNA-mediated carcinogenic signaling pathway network is intimately involved in glioma initiation and progression. In the present study, by performing experiments and bioinformatics analysis, we found that RPN2 was markedly elevated in glioma specimens compared with normal controls, and its upregulation was significantly linked to WHO grade and poor prognosis. Knockdown of RPN2 inhibited tumor proliferation and invasion, promoted apoptosis, and enhanced temozolomide (TMZ) sensitivity in vitro and in vivo. Mechanistic investigation revealed that RPN2 deletion repressed β-catenin/Tcf-4 transcription activity partly through functional activation of glycogen synthase kinase-3β (GSK-3β). Furthermore, we showed that RPN2 is a direct functional target of miR-181c. Ectopic miR-181c expression suppressed β-catenin/Tcf-4 activity, while restoration of RPN2 partly reversed this inhibitory effect mediated by miR-181c, implying a molecular mechanism in which TMZ sensitivity is mediated by miR-181c. Taken together, our data revealed a new miR-181c/RPN2/wnt/β-catenin signaling axis that plays significant roles in glioma tumorigenesis and TMZ resistance, and it represents a potential therapeutic target, especially in GBM.

## Introduction

Glioblastoma multiforme (GBM), as the most common primary tumor in adults, is highly heterogeneous and is correlated with genetic and epigenetic alterations. Although current molecular subtypes have been identified and applied to clinical treatment evaluation, such as isocitrate dehydrogenase (IDH) mutation, O6-methylguanine-methyltransferase (MGMT) promoter methylation, and 1p/19q loss. However, gene heterogeneity-mediated TMZ therapy resistance remains to be challenging^1,2^. Additionally, multiple aberrant gene and signaling pathways networks have been proved to be responsible for treatment resistance^3^. Hence, it is urgent to further identify relevant genes and complex regulatory networks, providing significant value for deeply understanding GBM molecular mechanisms and targeted treatment responses.

Early research indicated that glycosylation alterations are linked to GBM initiation and malignant progression^4,5^. Ribophorin II (RPN2) is highly conserved and is an essential part of the oligosaccharyltransferase complex, which is responsible for the N-glycosylation of multiple proteins^6^. Tominaga et al. demonstrated that RPN2 can facilitate the glycosylation of CD63, which subsequently induces multidrug resistance protein 1 (MDR1) glycosylation-mediated membrane translocation, leading to the breast cancer progression and drug resistance^7,8^. This type of RPN2-mediated glycosylation of p-gp (encoded by MDR1) is responsible for the treatment resistance of several tumors, including breast cancer, ovarian cancer, gastric cancer, and esophageal squamous cell carcinoma^9–12^. Mounting evidence indicates that RPN2 is a significant oncogene and plays a crucial role in cancer progression and drug resistance. However, the role of RPN2 in gliomagenesis and the relevant mechanisms have been poorly elucidated.

Wnt/β-catenin signaling is one of the main cancer pathways in GBM and represents a promising target for glioma therapy. β-Catenin, as a key effector, accumulates in the nucleus and interacts with the TCF/LEF family to promote the expression of specific oncogenes, including cyclinD1 and c-myc, whereas the GSK-3β/APC/Axin complex promotes its degradation under wnt inactivation^13,14^. Takahashi et al. demonstrated that RPN2 antagonizes GSK-3β through physical interaction leading to inactivation of GSK-3β phosphorylation and subsequently enhancement of mtp53 stabilization to promote the tumor initiation, metastasis, and cancer stem cell (CSC) property acquisition in breast cancer^15^. Therefore, based on the GSK-3β-mediated inhibitory role for the wnt/β-catenin pathway, we speculate that RPN2 may be implicated in GBM development and progression by regulating the GSK-3β/wnt/β-catenin pathway, which remains to be further experimentally verified.

Accumulating studies have confirmed that the miR-181 family is dramatically downregulated in glioma with different grades, and serves as an important tumor-suppressor in GBM initiation and progression^16,17^. Ayala-Ortega et al. reported that miR-181c is inactivated in glioblastoma cell lines due to DNA methylation at the miR-181c promoter region and to the dissociation of CCCTC-binding factor (CTCF), and verified that notch2 was a direct target by miR-181c^18^. In addition, miR-181c is significantly downregulated in GBM patients who respond to RT/TMZ, suggesting that miR-181c is involved in the chemoresistance. She et al. demonstrated that miR-181c enhances TMZ chemosensitivity by directly targeting Rap1B in GBM cells^19^. Furthermore, miR181c combined with miR-21, miR-128a, and MGMT methylation status can predict prognosis in GBM, and miR-181c and miR-21 predict 6-month progression with high sensitivity (92%) and specificity (81%)^20,21^. Although several miRNAs linked to the TMZ resistance through modulating the wnt/βcatenin pathway have been extensively identified, such as miR-155, miR-126^13,22^. However, there have been no relevant reports on miR-181c mediated wnt/β-catenin signaling inactivation in glioma.

In the present study, we confirmed that RPN2 is prominently upregulated in gliomas and is significantly associated with WHO grade and poor clinical prognosis. In addition, knockdown of RPN2 inhibited proliferation and invasion and sensitized GBM cells to the anticancer effects of the TMZ partly through suppression of the wnt/β-catenin signaling pathway. Moreover, we revealed that RPN2 is a direct functional target of miR-181c, while RPN2 restoration could reverse the facilitatory effect of miR-181c on TMZ sensitivity. Mechanistically, miR-181c significantly repressed wnt/β-catenin signaling, whereas this effect was abrogated by the restoration of RPN2. Hence, we, for the first time, revealed the critical regulatory role of RPN2 on miR-181c-mediated wnt signaling inactivation and TMZ sensitivity in glioma. Taken together, our results suggest that RPN2 may be a crucial prognostic factor, and that the miR181c/RPN2/wnt/β-catenin axis might act as a novel therapeutic target for glioma.

## Materials and methods

### Patients and samples

Gene expression data with relevant complete clinicopathologic variables consisting of GBM29 datasets and GBMLGG14 datasets were downloaded from the TCGA database (https://xenabrowser.net/datapages/). Additionally, 35 clinical glioma specimens were obtained from the nerve tumor tissues bank at the Tianjin Institute of Neurology, including 6 low grade (WHO I and II) and 29 GBM (WHO IV) samples according to the world WHO grading criteria. Five normal brain tissue samples came from patients with severe traumatic brain injury who needed post-trauma surgery. All the patients signed informed consent forms. This study was approved by the Research Ethics Committee of Tianjin Huan hu Hospital.

### Cell culture and transfection

The U251, U87, A172, LN229, and LN308 glioblastoma cells were obtained from the China Academia Sinica Cell Repository, Shanghai, China. T98G glioblastoma cells were established and characterized by the Laboratory of Neuro-Oncology of the Tianjin Neurological Institute. The low-grade glioma H4 cells were obtained from the Peking Union Medical College Cell Library. The cells were maintained in Dulbecco’s modified Eagle’s medium (DMEM, Gibco, USA) supplemented with 10% fetal bovine serum (FBS, Gibco, USA) and incubated at 37 °C with 5% CO_2_. The oligonucleotide sequences of human miR-181c mimic, RPN2 siRNA (sense:5′-GGAUCGCCCUUUCACAAAUTT-3′), GSK-3β siRNA (sense: 5′-GCUCCAGAUCAUGAGAAAGCUAGAU-3′), and pcDNA3.1-CMV-GFP-RPN2 overexpression plasmid, were artificially synthesized by GenePharma (Shanghai, China). A scrambled sequence was used as the negative control. The cells were transfected using X-tremeGENE transfection reagent (Roche, Germany) following the manufacturer’s protocol.

### Lentivirus infection and stable cell line establishment

The knockdown lentivirus vectors of RPN2 (LV16-RPN2-sh: 5′-GGATCGCCCTTTCACAAAT-3′) and LV16-NC were also purchased from GenePharma (Shanghai, China). After seeding the cells in 6-well plates and incubating for 24 h, cells were infected with sh-RPN2 or sh-NC at a multiplicity of infection (MOI) of 5 in the Opti-DMEM medium. To establish stable knockdown cell lines, infected cells were treated with puromycin (2 μg/ml for U251 and LN229 cells) for 7 days. The knockdown efficiency was evaluated by qRT-PCR and western blot.

### Total RNA extraction and real-time qPCR

Total RNA isolated from glioma tissues and cells was extracted from pretreated cells with TRIzol reagent (Invitrogen) and reverse-transcribed to complementary DNA (cDNA) for mRNA, including RPN2, c-myc, cyclinD1, TCF4 and AKT1 with PrimeScript RT Master Mix (Takara, Japan) according to the manufacturer’s instructions and GAPDH served as the internal control. To detect miR-181c, stem-loop RT was performed with a miScript PCR starter kit (Qiagen GmbH) according to the manufacturer’s instructions. qPCR was performed using miScript SYBR Premix Green PCR kit (Qiagen GmbH) and Roche LC480 quantitative Real-Time PCR system (Roche Diagnostics). U6 levels were selected as internal controls, and fold changes were calculated by the relative quantification (2^-△△Ct^) method. All the primers were listed in Supplementary Table S1.

### Dual-luciferase Reporter Assay

The 3′UTR of RPN2 containing miR-181c conserved binding sites and responding mutant sites were inserted into the pMIR-REPORT vector (Promega, USA). U251 and LN229 cells were cultured in 96-well plates and co-transfected with wild-type or mutant luciferase reporters and the miR-181c mimic or miR-NC using the X-tremeGENE transfection reagent (Roche, Germany). To evaluate the β-catenin/Tcf-4 transcriptional activity, we used the TOP-FLASH and FOP-FLASH luciferase reporter constructs. U251 and LN229 cells with or without RPN2 stable knockdown were transfected with 100 ng of a TOP-FLASH plasmid containing six TCF-binding motifs (Millipore) or 100 ng of a FOP-FLASH control plasmid containing that contained six mutated TCF-binding motifs (Millipore). To evaluate miR-181c mediated effect on wnt pathway via RPN2, U251, and LN229 were transfected with miR-181c mimic or co-transfection of miR-181c mimic and RPN2 overexpressing plasmid. Dual-luciferase activities were also detected 48 h after the transfection using the Dual-Luciferase Reporter Assay System (Promega), and the Renilla luciferase activity was used as an internal control.

### Western Blot, Immunohistochemistry, Immunofluorescence

The ExKine Total Protein Extraction Kit was purchased from Abbkine. Additionally, a DUALXtract Cytoplasmic and Nuclear Protein Extraction Kit (Dualsystems Biotech, Switzerland) was used to isolate cytoplasmic and nuclear proteins according to manufacturer’s protocol. Equal amounts of protein (30 μg/lane) were separated by 10% SDS‑PAGE and subsequently transferred to PVDF membranes (EMD Millipore).Western blot was performed using antibodies against RPN2 (1:200 dilution, Abcam), β-catenin, GSK-3β, cyclinD1, c-myc, TCF4, fibrillarin, GAPDH, β-actin (1:5000 dilution; Abcam), GSK-3β (phospho Ser9) (1:1000 dilution; Abcam), AKT1(1:2000 dilution, HuaAn Biotechbology).

For IHC and Immunofluorescence assays, detailed procedures or illustrations can consult the previous description^23^. The antibodies used for IHC were as follows: PCNA, Bcl2, and MMP2(1:1000 dilution, cell Signaling), Ki67, vimentin (1:100 dilution, ABclonal), and cleaved caspase 3 (1:500, Abcam). Immunofluorescence assays were performed using an antibody against β-catenin (1:200 dilution; Abcam).

### CCK-8, colony formation, invasion and apoptosis analysis

U251 and LN229 cells were cultured in 96-well plates with 3 × 10^3^ cells per well and transfected with siRPN2 or co-transfection of miR-181c and RPN2-overexpressing plasmid, respectively, after incubation for 24 h. Then, the cells were treated with indicated concentrations of TMZ (100,200 μM, Selleck) for another 48 h. Finally, cell proliferation and viability were evaluated by Cell-Counting Kit 8 (CCK-8, Dojindo, Japan) according to the manufacturer’s instructions. Half maximal inhibitory concentration (IC50) values were calculated via nonlinear regression, and the data were fitted to a sigmoidal dose-response relation using GraphPad Prism 6.0 software (GraphPad Software Inc. La Jolla, CA, USA). For the colony formation assay, the cells were seeded in 6-well plates (0.5 × 10^3^ cells/well) and cultured for 14 days. The resulting colonies were then washed twice with PBS, fixed with 4% formaldehyde for 10 min and stained for 30 min with 0.1% crystal violet (Sigma). The number of colonies was counted by ImageJ.

Corning Transwell insert chambers (8 µm pore size, Corning, Cambridge, USA) and BD Matrigel (BD Biosciences, USA) were used for the cell invasion experiment for different treatments of GBM cells. Cell apoptosis analysis was performed with the FITC Annexin V Apoptosis Detection Kit (BD Biosciences, USA) according to the manufacturer’s instructions, as previously described^24^.

### Xenograft model with nude mice

All animal protocols were performed under the approval of the Animal Care and Used Committee of Tianjin Huanhu Hospital. BALB/c-A nude mice at 4 weeks of age were purchased from the Animal Center of the Cancer Institute, Chinese Academy of Medical Science. First, they were randomly divided into 4 groups and every two groups were injected subcutaneously with RPN2 stable knockdown LN229 cells (lentiv-RPN2sh) and negative LN229 cells (lentiv-NC) (5×10^7^ cells in 150 µl), respectively. When all the groups formed the tumor at approximately 3 weeks, one of every two groups was injected intraperitoneally with TMZ or DMSO (30 mg/kg/day) every 3 days for another 2 weeks, and additionally, the tumor size was first calculated using the formula (Length × Width^2^/2), Then, the tumor size calculation was performed every 1 week, which was used to describe the tumorigenicity curve. At termination of the experiment, the mice were sacrificed, and tumor weight was calculated. Removed tumor tissues were subjected to further immunohistochemistry assays and TUNEL staining.

### Statistical Analysis

All statistical analyses were performed using GraphPad software version 6.0 (GraphPad Software, CA, USA) or IBM SPSS Statistics 23.0 (SPSS, Chicago, USA). Data are presented as the mean ± standard deviation of at least three independent experiments, and t-test and one-way ANOVA were performed to analyze statistically significant differences between two groups or multiple groups, respectively. The differences were considered to be statistically significant at *P* < 0.05. The χ^2^ test was utilized to evaluate the association between RPN2 expression and clinicopathological characteristics. The Kaplan–Meier method was used to evaluate the differences in survival rates, which were analyzed by the log-rank test. *p < 0.05, **p < 0.01, ***p < 0.001, ****p < 0.0001.

## Results

### RPN2 overexpression is associated with glioma grade and poor prognosis

**Fig. 1:**
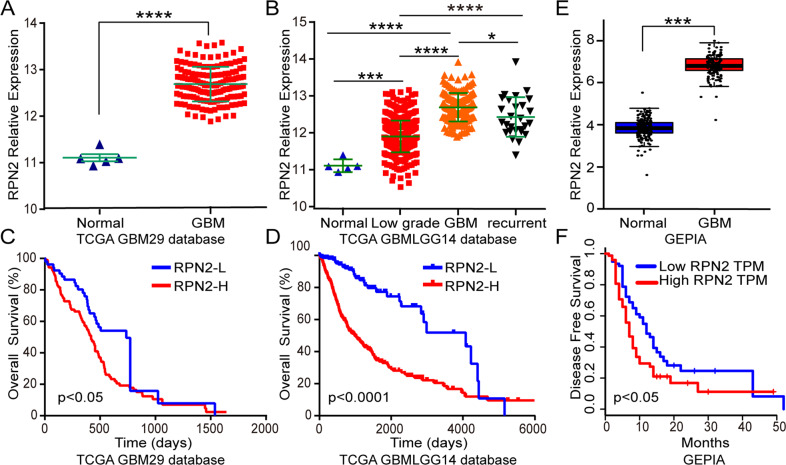
TCGA and GEPIA databases analyses indicate that increased RPN2 expression is positively associated with WHO grade and confers a poor prognosis in glioma patients. **a** RPN2 expression was analyzed in normal and GBM tissues of the TCGA GBM29 database. The significance of the differences was determined using Student’s t-test. **b** RPN2 expression was evaluated in normal, low grade, primary and recurrent GBM patients from TCGA GBMLGG14 database. Tukey’s test was used for multiple comparisons following ANOVA. **c**, **d** Kaplan–Meier survival curves for patients with low and high RPN2 expression from the TCGA29 database(c), TCGALGG14 database(d). *p*-value was determined using the log-rank test. **e** RPN2 expression analysis was also performed in normal and GBM tissues of the online GEPIA database. **f** Kaplan–Meier survival curves for patients with low and high RPN2 expression from the GEPIA database. High levels of RPN2 predicted a significantly worse outcome. *p < 0.05, **p < 0.01, ***p < 0.001, ****p < 0.0001.

**Fig. 2:**
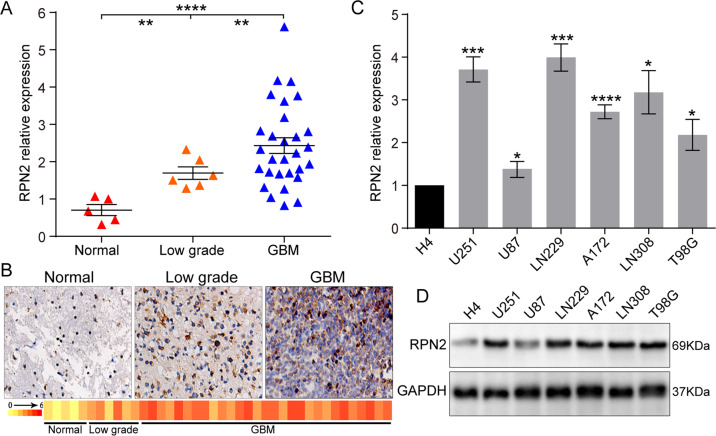
RPN2 is markedly upregulated in GBM cell lines and glioma specimens with different WHO grades. **a**, **b** Real-time PCR and immunohistochemical staining were performed to analyze RPN2 expression in 35 clinical specimens with various grades. **c**, **d** Real-time PCR and western blot analysis were used to analyze the RPN2 in GBM derived cell lines (A172, U251, U87, T98G, LN229, LN308) in comparison to the low-grade H4 cell line. GAPDH was used as an internal loading control. The significance of the differences between two groups was determined using Student’s t-test. *p < 0.05, **p < 0.01, ***p < 0.001, ****p < 0.0001.

To determine the expression profile of RPN2 in glioma clinical samples, we first analyzed two TCGA datasets, GBM 29 (containing 154 GBM samples and 5 normal tissues), and GBMLGG14 (containing 5 normal tissues, 516 lower grade, 161 GBM, and 27 recurrent samples), with different histological types. The analysis results showed that RPN2 was remarkably overexpressed in both primary and recurrent gliomas, especially primary GBM compared with the normal and lower grade samples (*p* < 0.0001) (Fig. 1a, b). In addition, RPN2 expression was positively associated with WHO grade. There was statistically significant difference between primary and recurrent gliomas, suggesting that RPN2 was linked to the glioma progression (Fig. 1b). Based on the relative complete survival data of the two datasets, the Kaplan–Meier survival curve method was utilized to analyze the relationship between RPN2 expression and overall survival. The patients were divided into low and high expression groups. The Kaplan–Meier survival curve analysis of the two datasets draw a unanimous conclusion that the RPN2 low expression group had a more prolonged survival than the RPN2 high expression group (Fig. 1c, d). Moreover, based on other important clinical parameters from the GBMLGG14 dataset, statistically significant relationships were observed between RPN2 expression and age, grade, IDH mutation, and histological type (Table 1). In addition, we also analyzed another public database named GEPIA, and the results showed that RPN2 was prominently upregulated in GBM and conferred to a poor prognosis (Fig.1e, f). Furthermore, the analysis from the CGGA database reached a consistent conclusion that RPN2 was evidently increased in WHO IV patients and positively related to WHO grade (Supplementary Fig. S1A), and RPN2 expression was linked to progression status (Supplementary Fig. S1B). The analysis of the prognostic significance from primary and recurrent gliomas also indicated that RPN2 overexpression predicted poor prognosis (Supplementary Fig. S1C, D). However, to tamp the RPN2 expression level and provide a solid foundation for future functional research, 35 surgically resected specimens were performed to further examine RPN2 expression by qRT-PCR and IHC, and the experimental results were consistent with the bioinformatics analysis (Fig. 2a, b). Besides, the western blot and RT-PCR analysis of 7 glioma cell lines found that RPN2 was upregulated in GBM cells compared with the lower grade glioma cell line H4 (Fig. 2c, d). All these data indicated that the RPN2 overexpression leads to a markedly worse outcome and may act as a key oncogene implicated in glioma initiation and progression.

**Table 1:** Relationship RPN2 expression and clinical parameters from TCGA GBMLGG database.

Variable	RPN2 low	RPN2 high	P*
**Gender**			0.483
Male	183	206	
Female	143	143	
**Age**			<0.001
<45	195	126	
≥45	130	223	
**Grade**			<0.001
Low	327	189	
High	0	161	
**IDH mutation**			<0.001
Yes	76	15	
No	14	20	
**Histological type**			<0.001
Astrocytoma	93	101	
Oligodendroglioma	233	88	
GBM	0	160	

P* was from χ^2^ test (two-sided).

### Silencing RPN2 can markedly inhibit glioma proliferation, invasion and enhance TMZ sensitivity

**Fig. 3:**
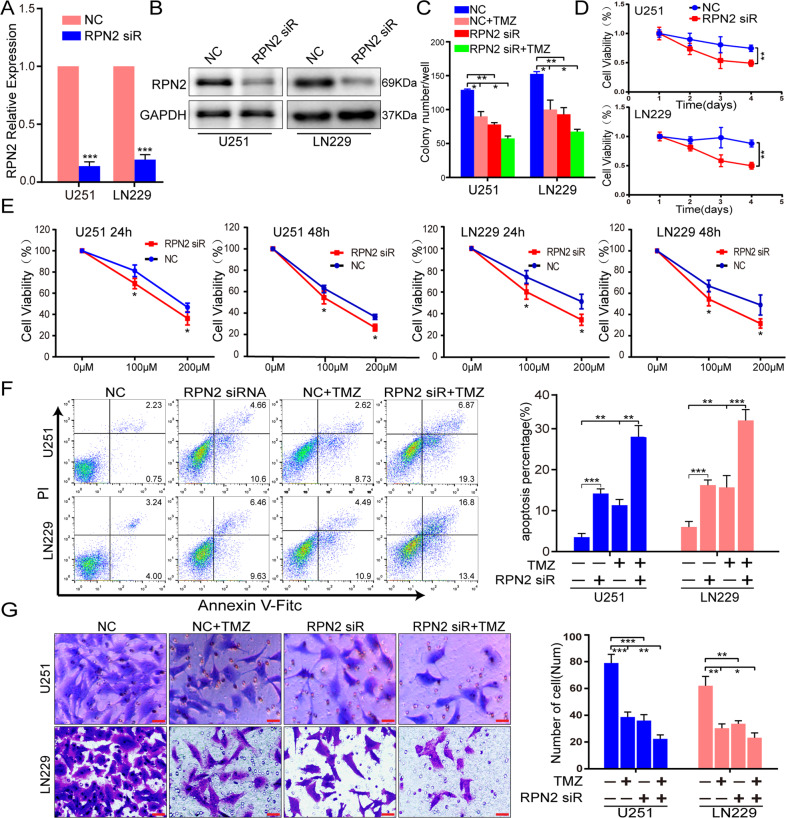
Knockdown of the RPN2 gene markedly inhibits proliferation and invasion, induces apoptosis and sensitizes U251 and LN229 cells to temozolomide. **a**, **b** Expression identification of RPN2 after knockdown via transfection with siRNA by real-time PCR(a) and western blot(b) in U251 and LN229 cells. **c** The U251 and LN229 cells were treated with TMZ or not at the concentration of 200 µM following the transfection with scramble (NC) or RPN2 siRNA, and cell proliferation ability was analyzed by colony formation assay 48 hours later. **d** CCK8 assay was performed to analyze the effect of RPN2siR on cell proliferation. **e** The U251 and LN229 cells were treated with different concentrations of TMZ solution (100 µM and 200 µM) following the transfection with scramble or RPN2 siRNA, CCK-8 assay was performed to evaluate TMZ sensitivity mediated by RPN2siR after 72 hours later, respectively. **f**, **g** Apoptosis and invasive ability of U251 and LN229 cells treated with TMZ (200 µM) or not following the transfection with scramble or RPN2 siRNA was examined by FITC annexin V (**f**) and Transwell methods (**g**), respectively. Scale bar, 100μm. *p < 0.05, **p < 0.01. The significance of the differences between two groups was determined using Student’s t-test. *p < 0.05, **p < 0.01, ***p < 0.001, ****p < 0.0001.

Based on explicit RPN2 upregulation both primary and recurrent gliomas, we explored the RPN2-mediated effect on the glioma malignant phenotype and the chemosensitivity to TMZ. According to RPN2 expression levels in different GBM cell lines, U251 and LN229 cells were selected for subsequent functional research. CCK-8, plate colony formation, and Annexin V FITC assays, were performed to examine cell proliferation, while the Transwell method was used to examine the invasive ability in U251 and LN229 cells with RPN2 knockdown via siRPN2 alone or with TMZ (200 μM) and siRPN2 co-treatment. The experiment contained four groups labeled NC, RPN2siR, TMZ and RPN2siR + TMZ. Knockdown by RPN2 siRNA was verified by real-time PCR and western blot (Fig. 3a, b). Compared with the NC group, knockdown of RPN2 markedly repressed the proliferative and invasive abilities (Fig. 3c, d, g) and promoted cell apoptosis (Fig. 3f) in the U251 and LN229 cell lines. Additionally, our findings revealed that the RPN2siR + TMZ group showed a lower proliferative and invasive capacity and a higher apoptotic percentage than the NC and TMZ groups, as displayed in Fig. 3c, e, g, f. In brief, our data suggest that downregulation of RPN2 can significantly attenuate the proliferation, and invasion, promoted the apoptosis, and enhance the chemosensitivity of U251 and LN229 cells to TMZ when comparing TMZ + siRPN2 with the TMZ group.

### Knockdown of RPN2 inhibits tumor growth and enhances chemosensitivity to TMZ in vivo

**Fig. 4:**
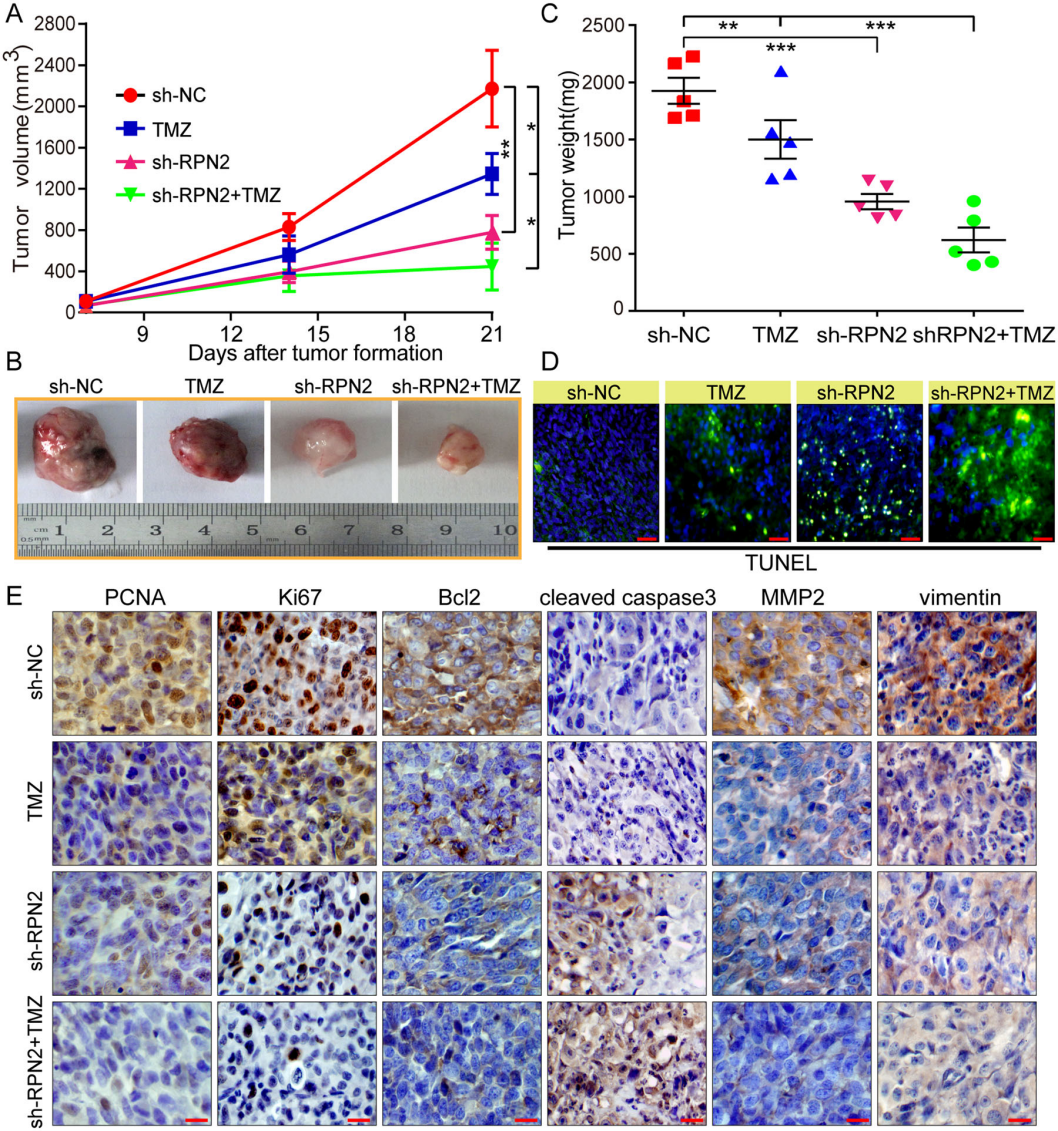
Subcutaneous tumor formation analysis and TMZ treatment experiment demonstrated that knockdown of RPN2 inhibits tumor growth and enhances TMZ chemosensitivity in vivo. LN229 cells stably expressing lentiv-shRPN2 or lentiv-NC were subcutaneously injected into nude mice (two groups per treatment). When almost all the mice had formed tumor (after 3 weeks), one of every two groups were intraperitoneally injected with TMZ (30 mg/kg/day) or DMSO (0.3%) every three days for another 3 weeks. **a** Tumor volume curves derived from LV-shRPN2 or LV-NC LN229 cells in the presence or absence of TMZ was monitored every week after tumor formation. **b** Representative images of excised tumors from different treatment group are shown. **c** Removed tumors weight from different group was evaluated at the endpoint. **d** TUNEL analysis from different treatment group was used to evaluate apoptosis in vivo. Scale bar, 50 μm. **e** Representative photomicrographs of immunohistochemistry of tumor sections for PCNA, Ki67, Bcl2, cleaved caspase 3, MMP2 and vimentin were illustrated in different groups. Scale bar, 50 μm. Data are presented as the mean ± S.D. *P* value was determined by one-way ANOVA. *p < 0.05, **p < 0.01, ***p < 0.001.

Given the RPN2-mediated inhibitory effect on the glioma malignant phenotype and TMZ sensitivity in vitro, we further performed an in vivo experiment using an LN229 xenograft model. A xenograft nude mouse model was established by LN229 cells or lentivirus-mediated RPN2 stable knockdown LN229 cells, respectively. After subcutaneous tumor formation for approximately 3 weeks for all groups, TMZ (30 mg kg/day/per mouse) and DMSO (0.3%) treatment every 3 days were further performed to treat one of the two groups (sh-NC and sh-RPN2 group), respectively. Tumorigenicity results revealed that the tumor formation rate of the NC group was markedly higher than that of the shRPN2 group within 2 weeks, and the tumor volume in the shRPN2 group was markedly smaller than that in the NC group until the terminate of the experiment (Fig. 4b). Additionally, TMZ treatment (TMZ group) retarded tumor growth compared with the NC group, and the group with treatment in combination with shRPN2 and TMZ (sh-RPN2 + TMZ) showed the smallest tumor volume in comparison to the other groups (Fig.4a, b). At the termination time of 3 weeks after the tumor formation in all groups, tumor weight was also evaluated. There was a statistically significant difference between the sh-RPN2 group compared with the NC group and the shRPN2+TMZ group compared with TMZ group, respectively (Fig. 4c). Consistent with the in vitro assay, the shRPN2+TMZ group showed the lightest tumor weight. Moreover, the apoptotic analysis by the TUNEL method was in accordance with the in vitro results (Fig. 4d). Furthermore, IHC results showed that knockdown of RPN2 decreased the expression of several indicators associated with cell proliferation, apoptosis and invasion, including PCNA, Ki67, Bcl2, MMP2, vimentin, and increased the cleaved caspase3 expression level, which was the most significant in shRPN2+TMZ group (Fig. 4e). In summary, these data indicate that the silencing of RPN2 can significantly repress tumor growth and sensitize tumors to TMZ in vivo, which was consistent with the in vitro assay results.

### Knockdown of RPN2 inhibits the wnt/β-catenin signaling pathway

**Fig. 5:**
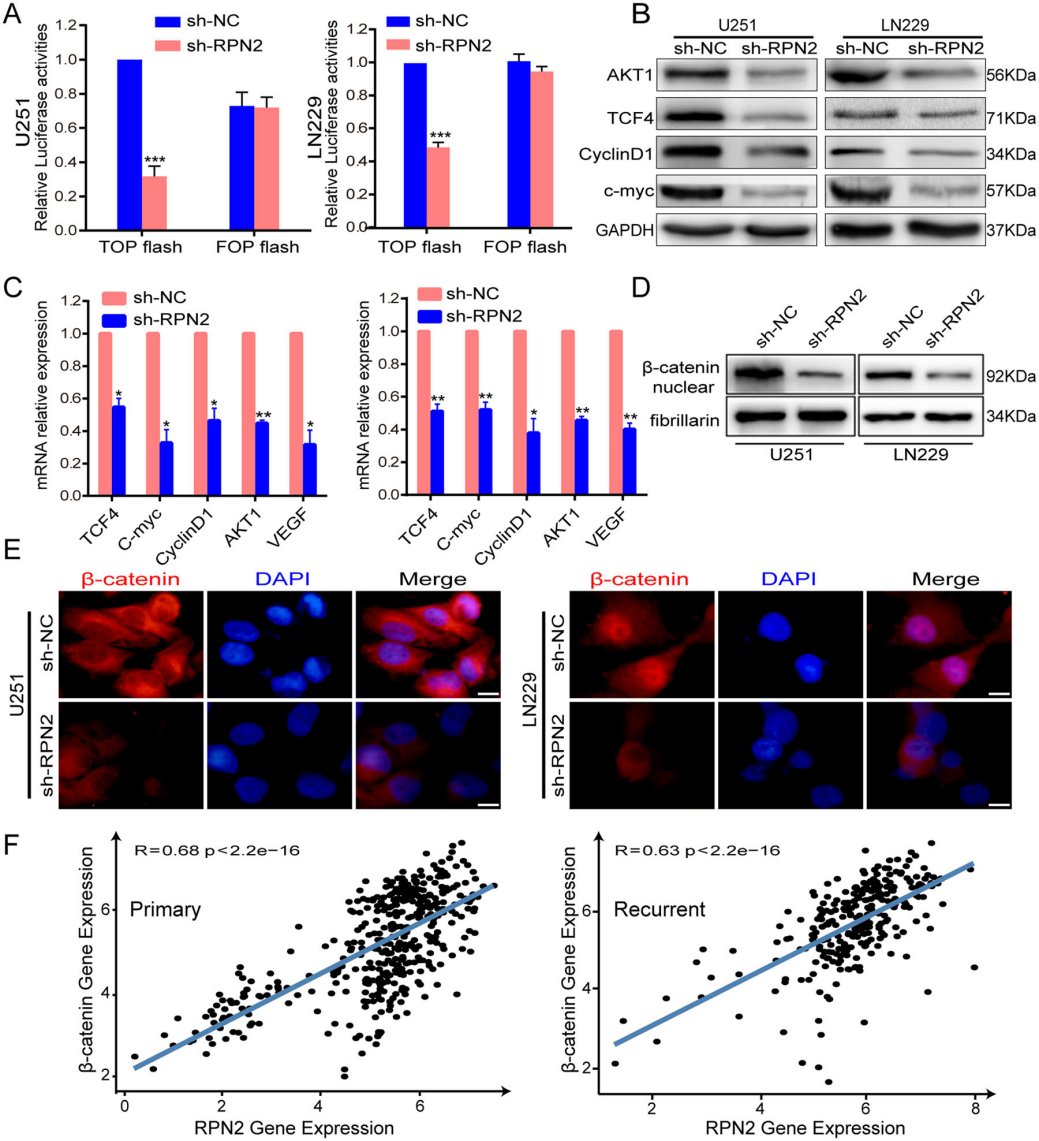
Knockdown of RPN2 inhibits β-catenin/TCF-4 transcriptional activity. **a** The U251 and LN229 cells stably expressing lentiv-shRPN2 or lentiv-NC were transfected with TOP/FOP plasmid and luciferase reporter assays were performed 48 h later. **b** Western blot was utilized to examine the expression level of wnt/β-catenin signaling downstream factors, including AKT1, TCF4, cyclinD1, c-myc in U251, and LN229 with or without stably knockdown of RPN2 mediated by lentiv-shRPN2. GAPDH was used as a loading control. **c** Real-time PCR analysis of wnt pathway downstream target genes containing TCF4, c-myc, cyclinD1, AKT1, and VEGF. **d** Western blot was utilized to detect the expression of nuclear β-catenin in lentiv-NC (sh-NC) and lentiv-RPN2(sh-RPN2) groups. Fibrillarin was used as a loading control. **e** Expression of β-catenin in the cytoplasm or nucleus was analyzed by immunofluorescence assay in U251 and LN229 cells with or without stably knockdown of RPN2 mediated by lentiv-shRPN2. **f** A significant positive correlation was identified between RPN2 and β-catenin expression from primary and recurrent gliomas of the CGGA database (Pearson’s correlation analysis). The data present as mean ± SD from three independent experiments. The significance of the differences between two groups was determined using Student’s t-test. *p < 0.05, **p < 0.01, ***p < 0.001.

Given that RPN2 can physically interact with GSK-3β and subsequently result in the inactivation of phosphorylation (Ser9) and that GSK-3β is a significant suppressor of the wnt signaling pathway, we further explored the relationship between RPN2 and wnt/β-catenin pathway, highlighting the detailed mechanism of shRPN2 in suppressing tumorigenicity in glioma. First, TOP/FOP FLASH luciferase assay was performed to evaluate the RPN2-mediated effect on β-catenin transcriptional activity, and the silencing of RPN2 (sh-RPN2) reduced Top with no change in FOP-FLASH luciferase activity (Fig. 5a). Western blot and qRT-PCR analysis revealed that the downstream targets of the wnt/β-catenin pathway, including c-myc, AKT1, clyclinD1 and TCF4, were markedly decreased when RPN2 expression was knocked down (Fig. 5b, c). Additionally, β-catenin expression in the nucleus was significantly reduced, as indicated by western blot and immunofluorescence assays (Fig. 5d, e). Furthermore, we analyzed the CGGA dataset and found that RPN2 expression was positively correlated with the β-catenin expression in both primary and recurrent gliomas (Fig. 4f). These data confirmed that shRPN2 significantly represses wnt/β-catenin pathway. However, we further explored whether RPN2 regulated the wnt pathway through GSK-3β, the western blot was used to detect the GSK-3β and p-GSK-3β(Ser9) expression level when knocking down the RPN2 expression. Indeed, sh-RPN2 significantly decreased p-GSK-3β(Ser9) expression and increased the GSK-3β level (Supplementary Fig. S2A), besides, the inhibition of GSK-3β by siRNA in U251 and LN229 cells stably knocking down RPN2 expression markedly reversed the shRPN2 mediated inhibitory effect for TCF4/β-catenin transcriptional activity by TOP/FOP luciferase assay (Supplementary Fig. S2B). Therefore, all the data suggest that RPN2 knockdown suppresses wnt/β-catenin signaling pathway, at least partially through GSK-3β activation.

### RPN2 is a direct target by miR-181c

**Fig. 6:**
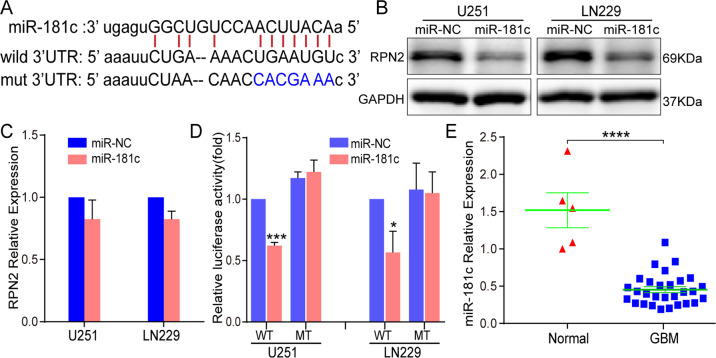
RPN2 is a direct functional target of miR-181c. **a** Schematic diagram of the seed sequence of miR-181c matching the RPN2 3′-UTR and the design of wild-type or mutant RPN2 3’-UTR containing reporter constructs. **b** RPN2 protein expression level analysis in U251 and LN229 cells transfected with miR-181c mimic or miR-NC 48 h later by western blot. GAPDH was used as an internal loading control. **c** RPN2 mRNA expression was examined in U251 and LN229 cells transfected with miR-181c mimic or miR-NC 48 h later by real-time PCR. **d** Luciferase reporter assays in U251 and LN229 cells, following co-transfection with the wild-type or mutant 3′UTR of RPN2 and miR-181c mimic. The data represent the fold change in expression (mean ± SE) of three replicates. **e** Real-time PCR was performed to evaluate miR-181c expression in 29 GBM patients compared with the normal brain tissues. The data present as mean ± SD from three independent experiments. The significance of the differences between two groups was determined using Student’s t-test. *p < 0.05, ***p < 0.001, ****p < 0.0001.

MiRNA-mediated regulation on mRNA at transcriptional and(or) post-transcriptional was one of the significant mechanisms responsible for gene inactivation. We used the TargetScan, MiRanda and PITA databases to identify the seed sequence of miR-181c that matched the 3′UTR of the RPN2 gene (Fig. 6a). Double luciferase reporter plasmid assay and western blot were performed to validate our prediction. The data revealed that RPN2 protein expression was significantly reduced compared with the mRNA expression level after upregulating miR-181c expression in U251 and LN229 cells (Fig. 6b, c). More importantly, luciferase reporter assay confirmed that upregulation of miR-181c led to a remarkable decrease of luciferase activity in pMIRWT RPN2 group, whereas no change of luciferase activity was examined in the pMIRMT RPN2 group (Fig. 6d). Besides, we also detected miR-181c expression levels in 5 normal and 29 GBM specimens, and miR-181c was dramatically downregulated in contrast to the expression tendency of RPN2 in GBM specimens (Fig. 6e). This evidence suggests that miR-181c modulates RPN2 expression by directly binding to the 3′-UTR of RPN2.

### MiR-181c can repress the wnt/β-catenin signaling pathway partially via RPN2

**Fig. 7:**
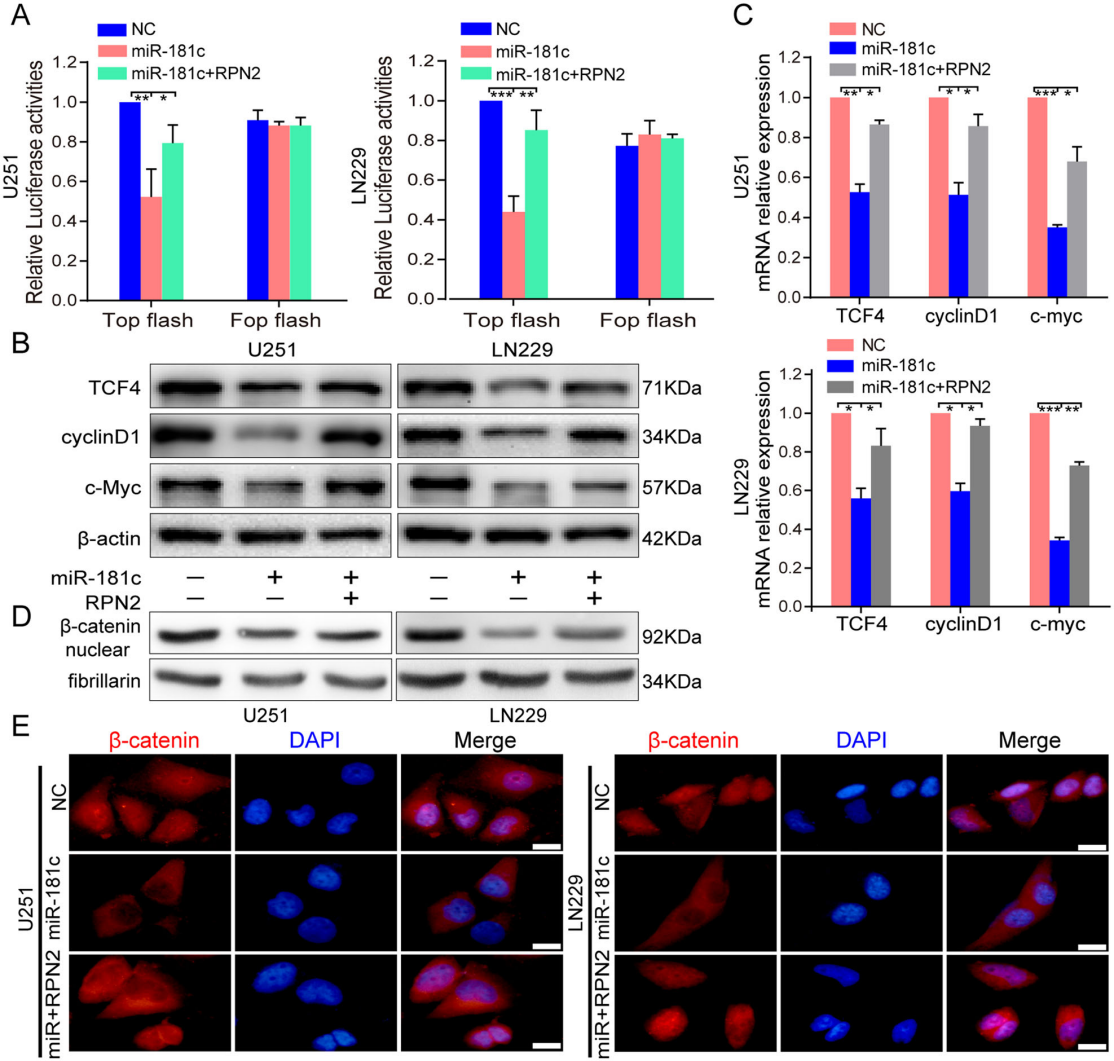
MiR-181c represses the β-catenin/TCF4 transcriptional activity, and restoration of RPN2 can reverse this effect by miR-181c. **a** TOP/FOP luciferase reporter assays were performed in U251 and LN229 cells transfected with TOP/FOP and miR-181c mimic or co-transfected with TOP/FOP, miR-181c mimic and RPN2 overexpressing plasmid. **b**, **c** Western blot and real-time PCR detection of wnt pathway downstream factors TCF4, cyclinD1 and c-myc expression level in U251 and LN229 cells transfected with miR-181c or co-transfected with miR-181c and RPN2. **d**, **e** Western bot and immunofluorescence assays were performed to examine the β-catenin expression in the nucleus and cytoplasm in U251 and LN229 cells transfected with miR-181c or co-transfected with miR-181c and RPN2, respectively. *p < 0.05. The data present as mean ± SD from three independent experiments. The significance of the differences between two groups was determined using Student’s t-test. *p < 0.05, **p < 0.01, ***p < 0.001, ****p < 0.0001.

Based on RPN2 being a direct functional target and RPN2-mediated regulatory effect on wnt pathway. we then explored whether miR-181c also regulates the wnt/β-catenin pathway via RPN2. Using the same experimental methods mentioned above, miR-181c ectopic expression markedly inhibited TOP activity with no apparent change in FOP activity by TOP/FOP luciferase analysis (Fig. 7a). Similarly, miR-181c could also dramatically suppress TCF4, c-myc and cyclinD1 expression by qRT-PCR and western blot (Fig. 7b, c), and β-catenin levels in the nucleus were markedly reduced following miR-181c upregulation by western blot and immunofluorescence assays (Fig. 7d, e). However, it was evident from the above data analysis that the restoration of RPN2 following the miR-181c mimic transfection can reverse the miR-181c-mediated inhibitory effect on wnt/β-catenin signaling (Fig. 7a–e). Taken together, these data suggest that miR-181c can attenuate wnt/β-catenin signaling, partially via RPN2, uncovering the existence of the miR-181c/RPN2/wnt pathway axis in GBM development and progression.

### RPN2 restoration can reverse the miR-181c-mediated enhanced effect on TMZ sensitivity

**Fig. 8:**
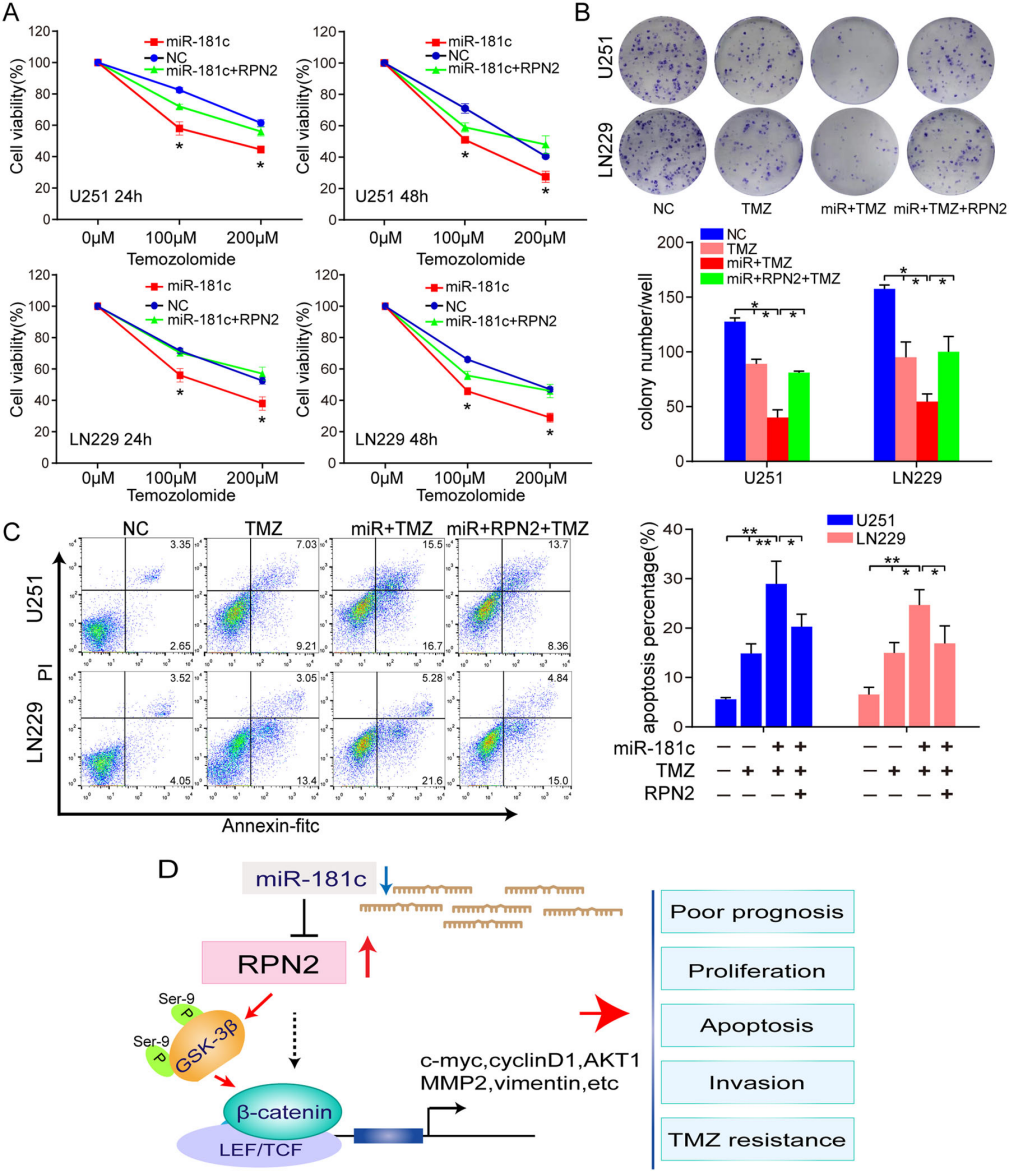
Restoration of RPN2 in miR-181c-overexpressing U251 and LN229 cells reverses miR-181c-mediated cell proliferation inhibition and apoptosis promotion. **a** U251 and LN229 cells transfected miR-181c mimic or co-transfected with miR-181c mimic and RPN2 overexpression plasmid were treated with TMZ at different concentrations (100 µM and 200 µM), and then CCK-8 assay was performed 24 and 48 hours later. **b** U251 and LN229 cells transfected miR-181c mimic or co-transfected with miR-181c mimic and RPN2 overexpression plasmid were exposed to 200 µM TMZ for 48 h and the proliferation was measured by colony formation assays. **c** Cells were transfected miR-181c mimic or co-transfected with miR-181c mimic and RPN2 overexpression plasmid and cultured in 200 µM TMZ, then subjected to apoptosis analysis by Annexin V/FITC assay. **d** A schematic diagram of the mechanism of the miR-181c/RPN2/wnt/βcatenin axis implicated in GBM progression and TMZ response. The data present as mean ± SD from three independent experiments. The significance of the differences between two groups was determined using Student’s t-test. *p < 0.05, **p < 0.01.

Previous studies have demonstrated that miR181c, acting as a tumor-suppressive factor, is closely related to TMZ chemosensitivity in glioma^18,19^. The present data have confirmed that RPN2 was a functional target of miR-181c. Hence, we hypothesized that the miR181c/RPN2/wnt axis might play essential roles in GBM drug resistance. Finally, we explored RPN2 function in the miR-181c mediated effect on TMZ sensitivity by CCK-8, colony formation and annexin V assay assays in U251 and LN229 cells. The results verified that the miR-181c markedly enhanced TMZ sensitivity, while RPN2 restoration via overexpression plasmids (absent 3’-UTR fragment) following transfection with miR181c mimic abrogated the effect on TMZ sensitivity mediated by miR-181c (Fig. 8a–c). Accordingly, these results reveal that miR-181c inhibits glioma proliferation and enhances TMZ chemosensitivity, at least partially via RPN2, highlighting that miR-181c/RPN2/wnt axis may play significant roles in GBM progression and TMZ resistance. A schematic diagram of the mechanism of the miR-181c/RPN2/wnt/βcatenin axis implicated in GBM progression is indicated in Fig. 8d.

## Discussion

Aberrant RPN2 gene overexpression has been documented to be frequently associated with multiple clinical parameters, including lymphatic metastasis, tumor grade, chemotherapy resistance and poor prognosis in a variety of tumors, including breast cancer, ovarian cancer, osteosarcoma, non-small-cell lung cancer, gastric cancer, laryngeal squamous cell carcinoma and colorectal cancer^6,10,25–29^, implying that RPN2 is a valid biomarker for drug response and is a potential therapeutic target. However, the studies of the role of RPN2 in glioma have rarely been reported. The bioinformatics analysis from the GEO database by Zhou et al. identified that RPN2 might be the significant gene among the top 10 hub genes acting as useful prognostic biomarkers for GBM^30^. In addition, Heroux et al. also reported that RPN2 was a significant biomarker of GBM using mass spectrometry-based label-free quantitative proteomics^31^. In our study, we first analyzed TCGA, CGGA and GEPIA databases and found that RPN2 was markedly upregulated in primary and recurrent GBM, which conferred a poor prognosis in glioma patients. Then, we also experimentally validated RPN2 overexpression in clinical specimens with various grades and GBM cell lines, and there was a positive between RPN2 expression and WHO grade, which was consistent with bioinformatics analysis. Therefore, we speculate that RPN2 might be a significant treatment target and prognostic factor for glioma, especially GBM.

GSK-3β is a key inhibitory factor of the wnt/β-catenin pathway by promoting phosphorylation and degradation of β-catenin, and the phosphorylation of GSK-3β at Ser9 results in its inactivation and subsequently enhancement of β-catenin trans transcriptional activity^32,33^. Besides, GSK-3β could directly degrade the wnt downstream target genes, c-myc and cyclinD1 via phosphorylation at different sites, and its dysregulation was closely related to the malignant progression of GBM^34^. Based on the report of RPN2 physical interaction and subsequent functional suppression in breast cancer, we explored role of RPN2 in the GSK-3β/wnt/β-catenin pathway. First, in combination with the TOP/FOP, qRT-PCR and immunofluorescence assays, we verified the molecular mechanism of sh-RPN2 mediated inhibition of wnt pathway. Furthermore, our data demonstrated that knockdown of RPN2 could attenuate p-GSK-3β(Se9) level and promote the GSK-3β functional activation, while the knockdown of GSK-3β reversed sh-RPN2 mediated inhibitory role for β-catenin transcription activity, as indicated in TOP/FOP assay, highlighting GSK-3β role in RPN2-mediated regulation for wnt/β-catenin pathway. However, the wnt pathway can form apparent crosstalk with PI3K/AKT/mTOR pathway to enhance glioma tumorigenicity^13^. AKT1 can facilitate inactivation of GSK-3β phosphorylation (Ser9) and enhance β-catenin nuclear translocation, while β-catenin could directly bind to the AKT1 promoter and regulate its expression at the transcriptional level^14^. In the present study, AKT1 expression was markedly reduced after knocking down the RPN2. Although the detailed mechanism of RPN2 mediated regulation for wnt/β-catenin pathway remains to be further elucidated, all the data indicate that RPN2-mediated GSK-3β/wnt/β-catenin/AKT1 loop network dysregulation plays critical roles in GBM progression and TMZ resistance.

RPN2-mediated glycosylation of P-gp (MDR1) is responsible for drug resistance in multiple malignancies^10,12^. Zhang et al. reported that RPN2 potentiates P-gp and ABCG2-mediated multidrug resistance via the ERK pathway in gastric cancer^11^. However, P-gp and ABCG2 were also significant molecular biomarkers linked to poor GBM prognosis and TMZ resistance^8^. Moreover, it was reported that activation of β-catenin by GSK-3β enhances the expression of p-gp through the MDR1 promoter in brain endothelial cells^35,36^. Riganti et al. demonstrated that TMZ decreases P‑gp expression in human blood–brain barrier cells by disrupting the Wnt3-mediated wnt/β-catenin pathway^37^. All these data highlighted the crucial role of the wnt pathway in RPN2-mediated TMZ resistance. However, whether the sh-RPN2 enhances TMZ sensitivity by attenuating β-catenin mediated transcriptional activation of P-gp remains to be further explored.

In addition to the wnt/β-catenin signaling pathway, numerous studies have proven that RPN2 promotes cancer progression by regulating various signaling pathways. For instance, Huang et al. demonstrated that RPN2 promoted metastasis and suppressed autophagy via STAT3 and NF-κB signaling pathways in hepatocellular carcinoma^38^. BI et al. also revealed the key role of the STAT3 pathway in RPN2 mediated colon carcinoma progression^39^. Besides, a recent study of glioma found that RPN2 repressed the radiosensitivity of glioma cells by activating STAT3 signal transduction^40^. Consequently, this evidence suggests that RPN2 may act as a significant oncogene and be involved in cancer progression and treatment resistance by activating a variety of carcinogenic signaling pathways.

It is well acknowledged that miRNA-mediated dysregulation of various mRNAs and related signaling pathways regulatory networks play key roles in regulating GBM progression, EMT, recurrence and TMZ resistance, such as the miR128/JAK2/C-JUN, miR21/PI3K/AKT and miR16/Bcl2 axis^41,42^. Then, we explored the miRNA-mediated upstream underlying mechanism of RPN2 gene inactivation. The report from Zhou et al. affirmed that miR-128 restrained cell proliferation and migration through the AKT-P53-cyclin pathway through directly targeting RPN2 in colorectal cancer^43^. By analyzing TargetScan, miRanda and PITA databases and performing subsequent experimental validation with double luciferase reporter plasmid assay and western blot, we verified that RPN2 is a direct functional target of miR-181c. Moreover, miR-181c dramatically suppresses the wnt/β-actenin pathway, while the restoration of RPN2 can partially reverse this inhibitory effect. Furthermore, RPN2 restoration can also effectively reverse the miR-181c-mediated enhancement of TMZ chemosensitivity. Accumulating evidence has demonstrated that miR-181c is involved in the suppression of GBM malignant progression and TMZ resistance through various target genes. MiR-181c can attenuate the malignant phenotype of GBM cells and enhance TMZ chemosensitivity by targeting Cdc42, RhoA, N-cadherin and Notch2^19,44^. He et al. indicated that miR-181c represses glioblastoma cell invasion and mesenchymal transition by targeting the TGF-β pathway^45^. The latest study demonstrated that exosome-derived miR-181c increases chemosensitivity by targeting MEST via the wnt/β-catenin signaling pathway in ovarian cancer^46^. However, our data uncovered the vital role of the RPN2/wnt/β-catenin axis in miR-181c mediated tumor suppression and TMZ sensitivity.

In conclusion, we, for the first time, revealed RPN2 upregulation in glioma tissues and GBM cell lines, which is negatively associated with clinical prognosis. In addition, we showed that knockdown of RPN2 dramatically attenuated GBM malignant progression and increased TMZ sensitivity in vitro and in vivo. Mechanistically, the silencing of RPN2 markedly inhibited wnt/β-catenin signaling pathway, at least partially dependent on GSK-3β activation. Furthermore, miR-181c mediated RPN2 inactivation by directly targeting the 3′-UTR of RPN2 in GBM. Overexpression of miR-181c enhanced TMZ sensitivity, partly via the RPN2/wnt/β-catenin signaling axis. Accordingly, our experimental findings validate the existence of the miR-181c/RPN2/wnt/β-catenin axis in GBM, and RPN2 may represent a potential therapeutic or combined treatment target for GBM.

## Supplementary information

Supplementary Figure legends

Supplementary Table. S1

Supplemantary Figure. S1

Supplementary Fig. S2
