# Supplementary material for: RPN2 is targeted by miR-181c and mediates glioma progression and temozolomide sensitivity via the wnt/β-catenin signaling pathway
Source: Cell Death Dis. 2020 Oct 22;11(10):890. doi: 10.1038/s41419-020-03113-5 (PMC7578010; doi:10.1038/s41419-020-03113-5)
Supplement: Supplementary file 1 — Supplementary Figure legends [file 41419_2020_3113_MOESM1_ESM.docx]

**Supplementary Fig. S1 RPN2 overexpression is associated with glioma grade and progression status, and predicts a poor prognosis according to the CGGA (Mseq693) analysis. a** RPN2 expression level analysis in gliomas with various grades according to the WHO grading criteria, indicating a significant positive correlation of expression with the WHO grade. **b** RPN2 expression analysis in primary and recurrent gliomas. **c, d** Kaplan–Meier survival curves for primary and recurrent gliomas with low or high RPN2 expression. High levels of RPN2 predicted a significantly worse outcome. **P*<0.05, ***p*<0.01, *****p*<0.0001.

**Supplementary Fig. S2 a** GSK-3β and p-GSK-β(Ser9) expression assays by western blot in U251 and LN229 cells stably expressing lentiv-sh-NC and lentiv-sh-RPN2. GAPDH was used as an internal loading control. **b** Real-time PCR analysis of GSK-3β after transfection with GSK-3β siRNA in U251 and LN229 cells stably knocking down the RPN2 expression or not (Left), and TOP/FOP luciferase activity analysis after knocking down the expression of GSK-3β by siRNA in U251 and LN229 cells stably expressing lentiv-sh-NC and lentiv-sh-RPN2 (Right). The data present as mean ± SD from three independent experiments. The significance of the differences between two groups was determined using Student’s t-test. **p*<0.05, ***p*<0.01.
