# Supplementary material for: RPN2 is targeted by miR-181c and mediates glioma progression and temozolomide sensitivity via the wnt/β-catenin signaling pathway
Source: Cell Death Dis. 2020 Oct 22;11(10):890. doi: 10.1038/s41419-020-03113-5 (PMC7578010; doi:10.1038/s41419-020-03113-5)
Supplement: Supplementary file 2 — Supplementary Table. S1 [file 41419_2020_3113_MOESM2_ESM.docx]

**Supplementary Table S1 Real-time PCR primer sequences**

| **Gene** | **Forward primer** | **Reverse primer** |
| --- | --- | --- |
| RPN2 | CTCTGACGCCCACTCACTAC | AATAGAGATCTTTGCATCTGGCAC |
| TCF4 | GGCTATGCAGGAATGTTGGG | GTTCATGTGGATGCAGGATAC |
| c-myc | GGCTCCTGGCAAAAGGTCA | CTGCGTAGTTGTGCTGATGT |
| cyclinD1 | CAATGACCCCGCACGATTTC | CATGGAGGGCGGATTGGAA |
| AKT1 | TCCTCCTCAAGAATGATGGCA | GTGCGTTCGATGACAGTGGT |
| VEGF | ATCTTCAAGCCATCCTGTGTGC | CTTTTAGGGACACCCGGAAC |
| GSK-3β | GGCAGCATGAAAGTTAGCAGA | GGCGACCAGTTCTCCTGAATC |
| GAPDH | CATGAGAAGTATGACAACAGCCT | AGTCCTTCCACGATACCAAAGT |
| MiR-181c | CCCATCTCAGCCTCCTAGT | GACCAACCTGAGCAACATAG |
| U6 | CTCGCTTCGGCAGCACA | AACGCTTCACGAATTTGCGT |
