# Supplementary figures and images for: RPN2 is targeted by miR-181c and mediates glioma progression and temozolomide sensitivity via the wnt/β-catenin signaling pathway
Source: Cell Death Dis. 2020 Oct 22;11(10):890. doi: 10.1038/s41419-020-03113-5 (PMC7578010; doi:10.1038/s41419-020-03113-5)

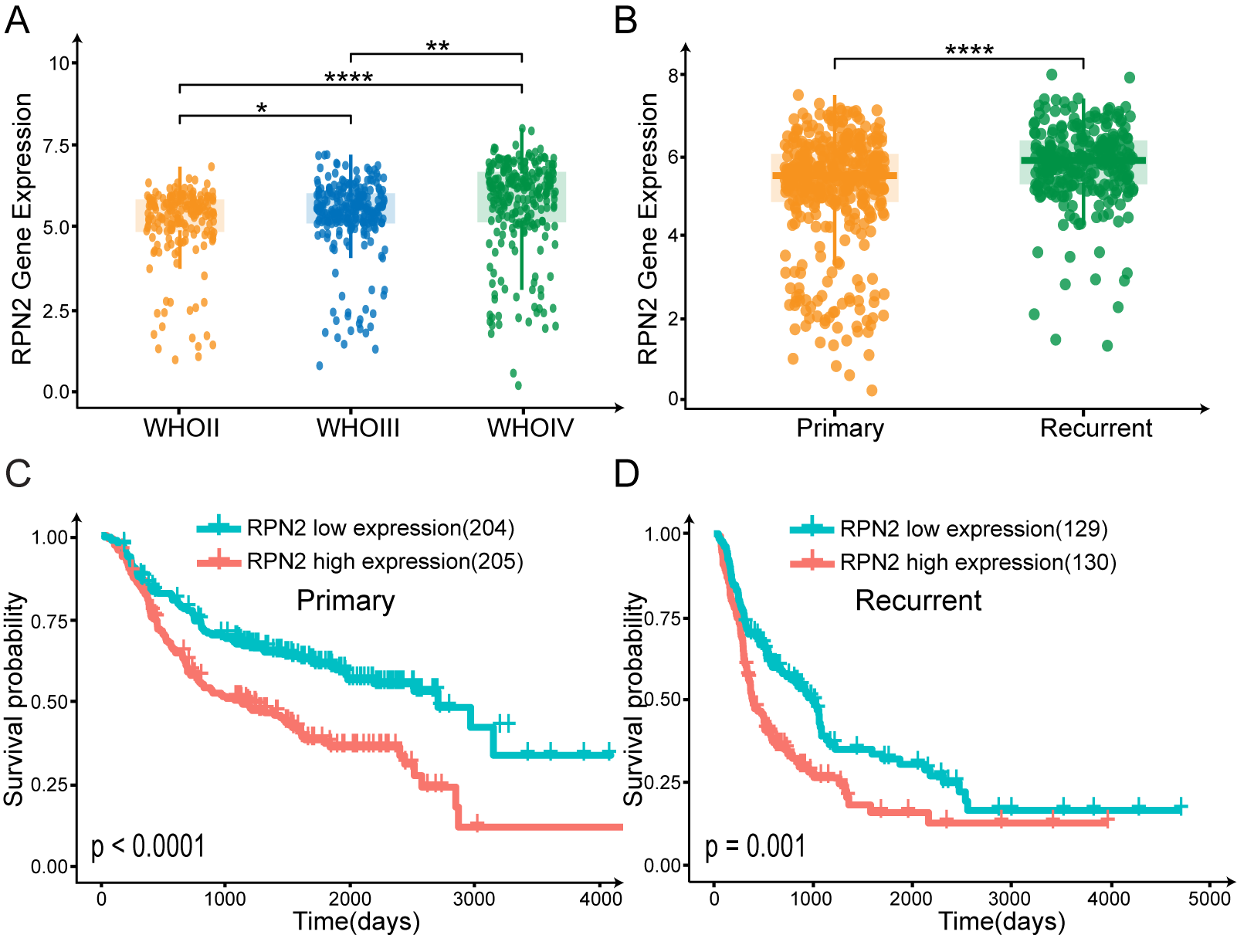

Supplement: Supplementary file 3 — Supplemantary Figure. S1 [file 41419_2020_3113_MOESM3_ESM.tif]

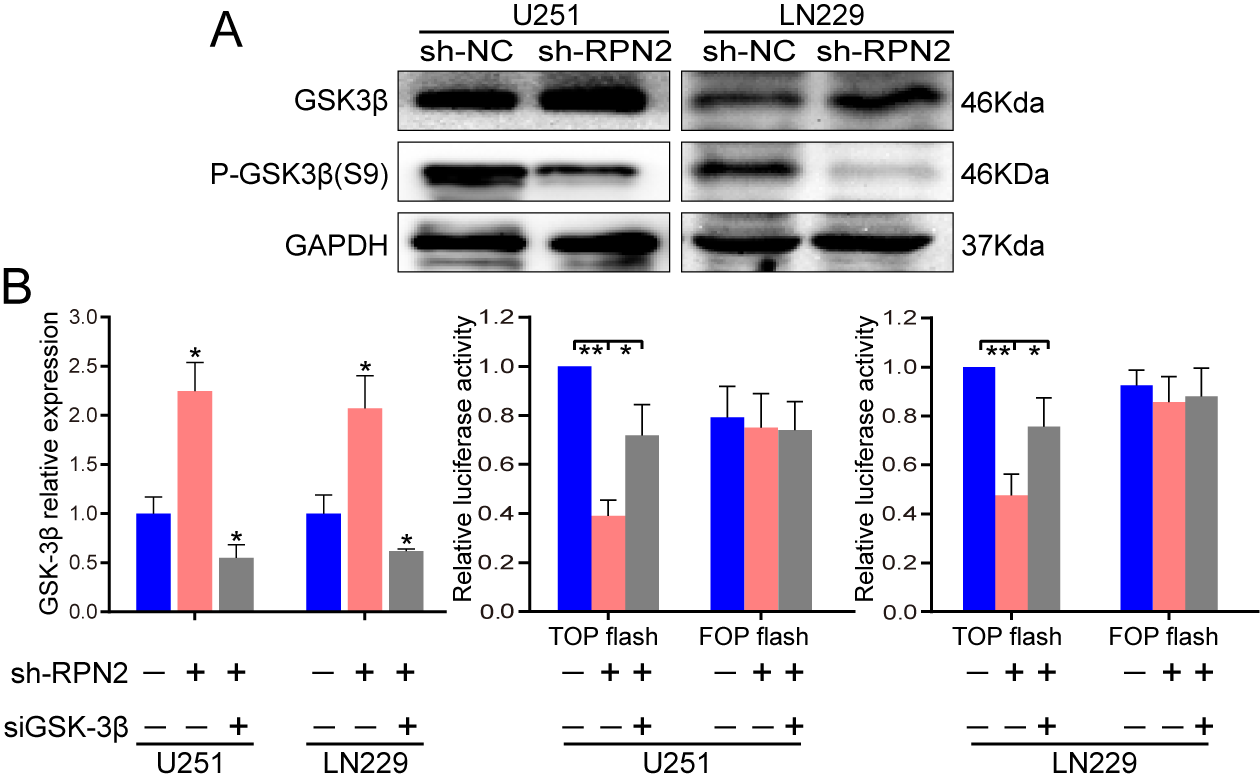

Supplement: Supplementary file 4 — Supplementary Fig. S2 [file 41419_2020_3113_MOESM4_ESM.tif]
